# AZU1: a new promising marker for infection in orthopedic and trauma patients?

**DOI:** 10.17179/excli2023-6705

**Published:** 2024-01-03

**Authors:** Philipp Hemmann, Lisa Kloppenburg, Regina Breinbauer, Sabrina Ehnert, Gunnar Blumenstock, Marie K. Reumann, Felix Erne, Johann Jazewitsch, Tobias Schwarz, Heiko Baumgartner, Tina Histing, Mika Rollmann, Andreas K. Nüssler

**Affiliations:** 1 Department of Traumatology and Reconstructive Surgery, BG Unfallklinik Tuebingen, Eberhard Karls University Tuebingen, Schnarrenbergstr. 95, 72076 Tuebingen, Germany; 2 Siegfried Weller Institute for Trauma Research, BG Unfallklinik Tuebingen, Eberhard Karls University Tuebingen, 72076 Tuebingen, Germany; 3 Department of Clinical Epidemiology and Applied Biometry, University of Tuebingen, Tuebingen, Germany

**Keywords:** AZU1, HBP, orthopedic surgery, infection, trauma surgery

## Abstract

Early and reliable detection of infection is vital for successful treatment. Serum markers such as C-reactive protein (CRP) and procalcitonin (PCT) are known to increase with a time lag. Azurocidin 1 (AZU1) has emerged as a promising marker for septic patients, but its diagnostic value in orthopedic and trauma patients remains unexplored. Between July 2020 and August 2023, all patients necessitating inpatient treatment for periprosthetic joint infection (PJI), peri-implant infection (II), soft tissue infection, chronic osteomyelitis, septic arthrodesis, bone non-union with and without infection were enrolled. Patients undergoing elective total joint arthroplasty (TJA) served as the control group. Blood samples were collected and analyzed for CRP, white blood cell count (WBC), PCT, and AZU1. Based on the inclusion and exclusion criteria 222 patients were included in the study (trauma = 38, soft tissue infection = 75, TJA = 33, PJI/II = 39, others = 37). While sensitivity and specificity were comparably high for AZU1 (0.734/0.833), CRP and PCT had higher specificity (0.542/1 and 0.431/1, respectively), and WBC a slightly higher sensitivity (0.814/0.455) for septic conditions. Taken together, the area under the curve (AUC) showed the highest accuracy for AZU1 (0.790), followed by CRP (0.776), WBC (0.641), and PCT (0.656). The Youden-Index was 0.57 for AZU1, 0.54 for CRP, 0.27 for WBC, and 0.43 for PCT. Elevated AZU1 levels effectively distinguished patients with a healthy condition from those suffering from infection. However, there is evidence suggesting that trauma may influence the release of AZU1. Additional research is needed to validate the diagnostic value of this new biomarker and further explore its potential clinical applications.

## Introduction

In the realm of sepsis diagnosis, the evaluation of over 100 promising biomarkers has yielded none-suitable for routine clinical use (Fisher and Linder, 2017[2]; Pierrakos and Vincent, 2010[12]). Recent years have witnessed the emergence of azurocidin 1 (AZU1), also known as heparin-binding protein (HPB) or cationic antimicrobial protein of 37 kDa (CAP37) (Naito et al., 2021[8]) as a promising serum biomarker for septic patients in intensive care units (ICU) (Fisher and Linder, 2017[2]; Sun et al., 2020[17]; Zhou et al., 2019[20]). 

AZU1 exhibits early release in response to bacterial antigens and is located in azurophilic granules and secretory vesicles of neutrophils (Fisher and Linder, 2017[2]; Snall et al., 2016[16]; Tapper et al., 2002[18]). This rapid response capability positions AZU1 as a valuable marker for the early stages of infection, activating various cell types and modulating the inflammatory response (Fisher and Linder, 2017[2]). 

Procalcitonin (PCT), a well-established marker for diagnosing bacterial infections (Hamade and Huang, 2020[6]; Sigmund et al., 2021[15]), correlates with infection severity and reaches its peak approximately six hours after sepsis onset (Sigmund et al., 2021[15]). Although PCT outperforms -C-reactive protein (CRP) and white blood cell count (WBC), it is neither sensitive nor specific enough to serve as a stand-alone marker (Downes et al., 2020[1]). Additionally, viral infections do not elicit the same elevations in PCT levels as bacterial infections (Downes et al., 2020[1]; Hamade and Huang, 2020[6]).

CRP, widely employed for diagnosing and monitoring infections, trauma, and other inflammatory conditions, offers accessibility, speed and affordability (Plebani, 2023[13]). However, its low specificity often leads to false-positive results (Sigmund et al., 2021[15]).

Both PCT and CRP are triggered only in response to infectious stimulation, causing a delay compared to AZU1. AZU1 holds promise as a serum biomarker for early infection detection and successful treatment. Zhou et al., conducted a prospective study with septic ICU patients, comparing AZU1 with PCT and CRP and support AZU1's potential (Zhou et al., 2019[20]). 

To date, no published study has explored AZU1 as an early marker in orthopedic and trauma patients with suspected infection. This study's primary objective is to evaluate the diagnostic suitability and accuracy of AZU1 as a serum biomarker for detecting infections in comparison to PCT, WBC, and CRP.

## Materials and Methods

### Patient population

Between July 2020 and August 2023, patients (n = 222) who presented at our level one trauma care emergency department and subsequently received inpatient treatment for confirmed or suspected periprosthetic joint infection (PJI), peri-implant infection, or soft tissue infection were included in this study. Due to initial suspicion of infection, patients with chronic osteomyelitis, septic arthrodesis, and bone non-union who required inpatient treatment were also included.

The control group (n=71) comprised two sets of patients: one group consisting of individuals scheduled for planned total joint arthroplasty (TJA), with a total of 33 participants, and another group consisting of 38 trauma patients.

Inclusion criteria encompassed individuals aged 18 or older who provided informed consent and were scheduled for either trauma or orthopedic surgery. Exclusion criteria implied individuals under the age of 18, those with cognitive impairment, those with missing blood samples, those undergoing non-surgical therapy, or those who did not provide informed consent. 

Blood samples and processing procedures were conducted as follows: WBC and CRP were determined in each patient during hospital admission. AZU1 and PCT were determined on the same day or one to three days later. Each patient provided a total of 7 ml of venous blood. Of this, 2.7 ml were collected in EDTA K3E tubes, while the remaining 4 ml were collected in serum CAT-tubes. These samples were stored in a refrigerator at 4-8 °C for not more than 60 minutes. Subsequently, the samples underwent centrifugation for ten minutes at 1000 g using the Thermo Scientific TM Heraeus Megafuge 40 Universal Centrifuge (Thermo Fischer Scientific, Dreieich, Germany). Following centrifugation, pipetting was performed, resulting in three 450 µl EDTA plasma samples and three 450 µl serum samples. These samples were then stored at -80 °C in a Thermo ScientificTM FormaTM 900 Series freezer (Thermo Fischer Scientific, Dreieich, Germany).

Quantitative analysis of AZU1 (≥15 ng/ml) and PCT (≥100 pg/ml) was carried out by ELISA, with kits from RayBio® (ELH-AZU1) for AZU1 and kits from R&D Systems® (DY8350-05) for PCT. WBC levels (within the range of 4,000-10,000/µl) and CRP levels (>5 mg/l) were determined as part of the standard blood draw upon hospital admission and after surgery in the in-house laboratory.

Furthermore, we conducted a comprehensive search in the hospital database to identify intraoperatively obtained microbiological samples.

For statistical analyses, JMP® (SAS Institute Inc., 16.2.0, Cary, NC, USA), Microsoft© Office Excel 2016 (Microsoft Corporation, Redmond, USA), and GraphPad Prism 8.0.1 (GraphPad Software Inc., Boston, MA, USA) were used. The significance level was set at p ≤ 0.05, with the removal of blood sample outliers when necessary according to the ROUT method (robust regression followed by outlier identification). The Mann-Whitney test was applied for non-parametric two-sample comparisons, while the Kruskal-Wallis test was used for comparisons with multiple groups including a control group. Receiver operating characteristics (ROC) were calculated to assess the diagnostic performance of various biomarkers based on the TJA group. This included computating the area under the curve (AUC), 95 % confidence interval (CI), sensitivity, specificity, and the Youden Index for diagnostic accuracy (Youden, 1950[19]).

The study protocol was approved by the ethics committee (346/2015BO2) of the local University.

## Results

**Figure 1:**
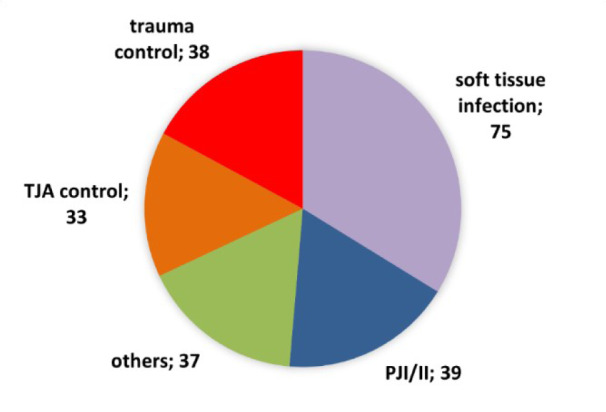
Cohort sizes of each group: TJA = total joint arthroplasty, PJI/II = periprosthetic joint infection/implant infection; “others” including patients with chronic osteomyelitis, septic arthrodesis, pseudarthrosis with and without infection

A total of 222 patients were enrolled and categorized into five distinct groups, as illustrated in Figure 1(Fig. 1). The most substantial group was the one with the soft tissue infection (STI) cohort, comprising 75 patients, closely followed by the PJI and implant infections (PJI/II) group with 39 patients. Additionally, 38 patients were assigned to the trauma control group, while the TJA cohort included 33 patients. The cohort “others”, consisting of 37 patients, encompassed individuals with such conditions as chronic osteomyelitis, septic arthrodesis, non-union with and without confirmed infection. These patients could not be matched to any other cohort and were therefore assigned to a separate cohort. The patient characteristics and distribution of microbiological information within each group are shown in detail in Table 1(Tab. 1).

**Figure 2:**
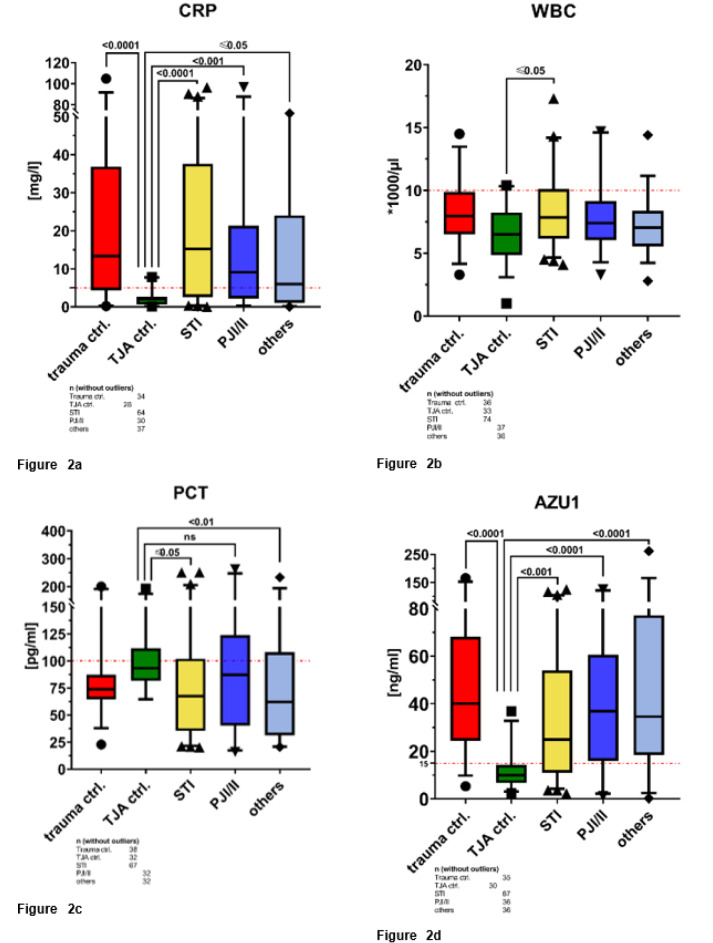
(a) Boxplots for CRP, TJA= total joint arthroplasty, STI= soft tissue infection, PJI/II= periprosthetic joint infection/implant infection. The highest CRP levels were found in the trauma and STI cohort. All cohorts showed a significantly higher CRP level than the TJA cohort. Red dashed line quantifies the designated threshold (<5 mg/l). (b) Boxplots for WBC, TJA= total joint arthroplasty, STI= soft tissue infection, PJI/II= periprosthetic joint infection/implant infection. No group was above the threshold of >10,000 WBC/µl. The STI cohort showed a significantly higher WBC compared to the TJA group. All other groups showed no statistically significant difference. Red dashed line quantifies the designated threshold (>10,000/µl). (c) Boxplots for PCT, TJA= total joint arthroplasty, STI= soft tissue infection, PJI/II= periprosthetic joint infection/implant infection. The TJA cohort showed the highest mean PCT level. No group exceeded above the threshold. The STI group and the cohort “others” were significantly lower than the TJA cohort. Red dashed line quantifies the designated threshold (>100 pg/ml). (d) Boxplots for AZU1, TJA= total joint arthroplasty, STI= soft tissue infection, PJI/II= periprosthetic joint infection/implant infection. All groups, except the TJA control, were above the estimated threshold of 15 ng/ml. The TJA group showed significantly lower AZU1 levels than all other cohorts

CRP blood values obtained after admission indicated significantly higher levels in all cohorts compared to the TJA group (trauma group = p<0.0001, STI = p<0.0001, PJI/II = p<0.001, others = p≤0.05, see Figure 2a(Fig. 2)). The highest CRP levels were found in the trauma and STI cohort. The PJI/II were higher than the cohort “others”. All groups were above the threshold of <5 mg/l except the TJA cohort. The initial WBC count following admission was also significantly elevated in patients with STI in comparison to the TJA group (p≤0.05), as depicted in Figure 2b(Fig. 2). However, it is important to note that none of the groups exceeded the established threshold for this marker. A similar pattern was observed for PCT, as illustrated in Figure 2c(Fig. 2). Interestingly, PCT values showed a high variance between the groups with the highest mean level for the TJA cohort. The STI cohort and the cohort “others” showed significantly lower PCT levels than the TJA control with p≤0.05 and p<0.01, respectively. All groups, except the TJA control, surpassed the designated AZU1 threshold of 15 ng/ml, as highlighted by the red dashed line in Figure 2d(Fig. 2). Furthermore, AZU1 levels were significantly higher in all groups when compared with the TJA control (trauma group = p<0.0001, STI = p<0.001, PJI/II = p<0.0001, others = p<00001).

ROC curves were calculated to determine diagnostic accuracy for each marker. 

**Figure 3:**
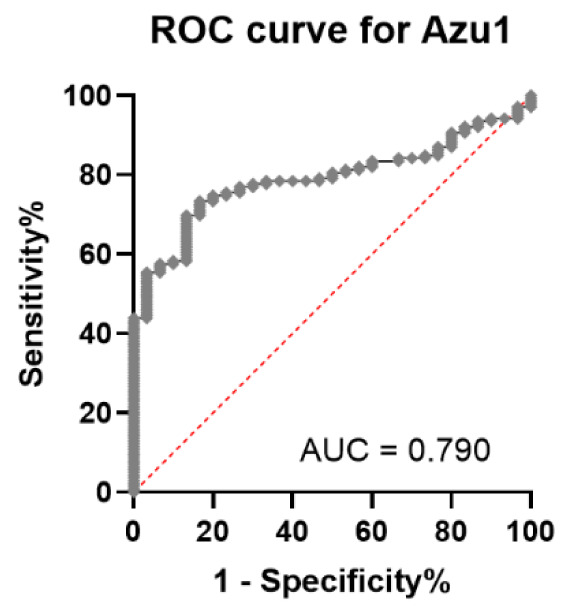
Receiver operating characteristics (ROC) curve for AZU1 (AUC=0.790)

**Figure 4:**
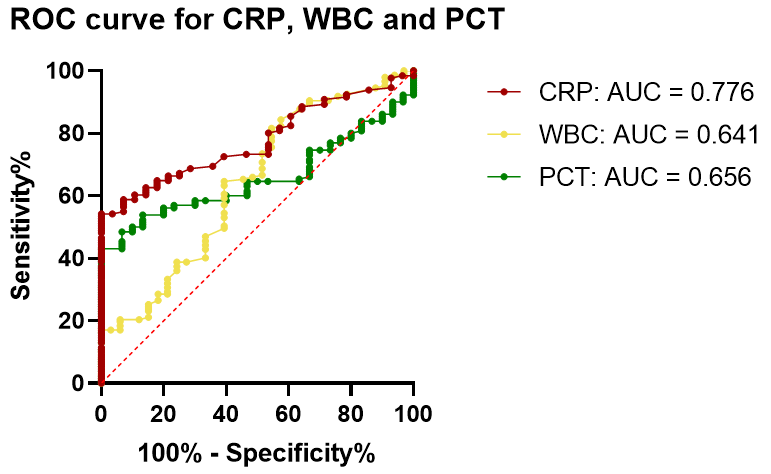
Receiver operating characteristics (ROC) curve for CRP (AUC=0.776), WBC (AUC=0.641), PCT (AUC=0.656)

The area under the curve (AUC) for AZU1 stood out as the highest at 0.790 when compared to the other markers, as evident in Figures 3(Fig. 3) and 4(Fig. 4). With a calculated threshold set at >16 ng/ml, AZU1 exhibited a sensitivity of 0.734 and a specificity of 0.833. Notably, the Youden index for AZU1 was the most favorable at 0.57 defining the threshold to be most favorable at >16 ng/ml. On the other hand, WBC displayed the highest sensitivity at 0.814 but had a relatively lower AUC of 0.641 and a Youden Index of 0.27. CRP presented the second highest AUC with 0.776. Sensitivity and specificity were 0.542 and 1, respectively with a threshold set at >8.05 mg/l. The Youden-Index was slightly lower than that of AZU1 at 0.54. PCT revealed the lowest AUC with 0.656. Sensitivity was the lowest with 0.431 compared to the other markers. The Youden-Index was 0.43. The diagnostic accuracy for all markers is summarized in Table 2(Tab. 2).

Furthermore, diagnostic accuracy was recalculated for all positive microbiological samples, regardless of the group. This analysis aimed to assess the diagnostic value of each biomarker independently of the specific disease entity, as illustrated in Table 3(Tab. 3). Notably, the AUC for AZU1 increased to 0.810 in this context. Similarly, the AUC for all other markers also raised, accompanied by an enhancement in their respective Youden indices. AZU1's sensitivity increased to 0.761, whereas specificity remained unchanged with 0.833. The AUC of CRP also increased to 0.806 as well as the sensitivity to 0.624 with a new threshold set at >8.3 mg/l. The AUC of WBC also enhanced to 0.655 with an increase of its sensitivity of 0.857 at a lower threshold of >5,450/µl. However, the specificity decreased slightly to 0.424. AUC of PCT increased to 0.661. Sensitivity slightly improved to 0.458 with a threshold that remained the same. Specificity did not change.

## Discussion

This study is the first assessment of the diagnostic potential of AZU1 as a novel marker for patients suffering from infections in orthopedic and trauma surgery.

AZU1 is currently used primarily for sepsis diagnosis in ICU patients in China (Sun et al., 2020[17], Zhou et al., 2019[20]). Zhou et al. conducted a study involving 125 patients with suspected sepsis and 56 healthy controls, evaluating CRP, PCT, and AZU1 levels (Zhou et al., 2019[20]). They found that AZU1 levels significantly differed among patients with septic shock (153.8 ng/mL), sepsis without shock (49.7 ng/mL), and local infections (11.8 ng/mL) (Zhou et al., 2019[20]). Notably, AZU1 exhibited a higher AUC of 0.893, outperforming PCT and CRP with AUCs of 0.856 and 0.699, respectively, in differentiating sepsis from infections. The accuracy for distinguishing septic shock from sepsis patients declined slightly, with an AZU1 AUC of 0.760. In our present study, AZU1 demonstrated an AUC of 0.790 when comparing all infected groups, rising to 0.810 in cases with positive microbiological samples. Interestingly, contrary to Zhou et al.,'s findings of lower AZU1 levels in patients with local infections (Zhou et al., 2019[20]), our study revealed significantly higher AZU1 levels across all cohorts when compared to the healthy TJA control group. This discrepancy could be attributed to the unique storage of AZU1 in vesicles, allowing for immediate release at the onset of infection. Unlike PCT or CRP, AZU1 does not require a trigger-based biosynthesis, enabling earlier diagnosis. However, it's worth noting that AZU1 levels may be erroneously low in patients with leucopenia (Funke et al., 2000[3]). As indicated by Zhou et al., three of the reported five sepsis patients with leucopenia had AZU1 levels below the cutoff value (Zhou et al., 2019[20]).

Furthermore, the trauma group exhibited elevated AZU1 levels (refer to Figure 2d(Fig. 2)) despite their lack of infection. This suggests that trauma itself may trigger the release of AZU1. Halldorsdottir et al. found significantly higher AZU1 levels in patients with a high Injury Severity Score, shock on arrival, or massive transfusion (Halldorsdottir et al., 2018[5]). Notably, the increase in CRP due to trauma is a well-established clinical phenomenon (Giannoudis et al., 2004[4]) which our study also confirmed. In an additional sub-analysis according to Spearman correlation, we observed a significant positive correlation between AZU1 and CRP in trauma patients (ρ=0.5333; p=0.0029). The same sub-analysis was performed within the TJA cohort. No significant correlation could be found between CRP and AZU1 (ρ=-0.0712; p=0.735) which underlines that trauma or fracture may release AZU1. However, the clinical significance of this correlation remains uncertain, primarily due to the limited size of the trauma cohort comprising only 38 patients.

Omar et al. conducted a study evaluating PCT as an early biomarker for infected diabetic foot ulcers (Omar et al., 2023[9]). They compared 50 diabetic patients without ulcers, 107 patients with non-infected diabetic foot ulcers, and 107 with infected ulcers. Their results indicated an elevated mean level of PCT in the infected diabetic foot ulcer group compared to the other two groups. However, the study reported relatively low sensitivity (63.6 %) and specificity (83.2 %) for PCT, leading to the conclusion that PCT is insufficient for early diagnosis of infected diabetic foot ulcers (Omar et al., 2023[9]). Notably, the study's limited sensitivity may be attributed to the absence of septic patients or those with septic shock, who typically require ICU admission. PCT is known to be associated with the severity of infection (Ozbay et al., 2023[10]) whereas all patients in this study presented with local signs of infection.

Of particular importance is the substantial distinction observed between the TJA group and the PJI/II cohort. Physicians face challenges when diagnosing periprosthetic joint infection (PJI) in patients with atypical clinical symptoms that do not align with common PJI definitions by Musculoskeletal Infection Society (MSIS), International Consensus Meeting (ICM) or European Bone and Joint Infection Society (EBJIS) (McNally et al., 2021[7]; Parvizi et al., 2018[11]; Shohat et al., 2019[14]). AZU1 could potentially serve as an additional marker to enhance diagnostic accuracy. Its implementation can be easily integrated into clinical routine. AZU1 can be acquired during the standard blood withdrawal and subsequently be determined by ELISA at in-house laboratory. At present, our hospital outsources the determination of synovial alpha-defensin to an external laboratory, resulting in increased logistical effort and costs. Additionally, the results are delivered with a delay of several days. Consequently, further comprehensive studies are necessary.

It is important to note that there are several limitations to this study that need to be mentioned. First, the cohorts were relatively small, heterogeneous and not randomized, potentially impacting accuracy, robustness and reproducibility of AZU1 validation. Second, AZU1 evaluation occurred at a single time point during the disease course, overlooking potential fluctuations. Additionally, some patients had prior treatments and chronic infections, further complicating the analysis. Last, variations in the timing of blood sample collection should be considered as well, since CRP and WBC were obtained upon hospital admission, while PCT and AZU1 sampling took place later, following informed consent. Consequently, some patients did not undergo AZU1 and PCT testing until the subsequent day or prior to surgery, depending on admission time, including overnight admissions.

## Conclusion

In conclusion, this pioneering study represents the first investigation into AZU1 as an infection marker for orthopedic and trauma patients. While AZU1 exhibited the capacity to detect infections, its precise value as an additional diagnostic parameter needs to be clarified. Notably, trauma appears to stimulate AZU1 release. For patients with PJI or implant infections (II), AZU1 may offer another valuable tool for early detection of implant-related infections. Validation of this marker's suitability and utility requires additional comprehensive and larger-scale studies.

## Declaration

### Author's contribution

PH: interpretation of the results, writing and revising the manuscript

LK: data collection, statistical analysis, figures

RB: data collection, statistical analysis, supervision, revising the manuscript

SE: revising the manuscript

GB: statistical consulting, revising the manuscript

MKR: revising the manuscript

FE: revising the manuscript, literature research

JJ: revising the manuscript, figures

TS: biomedical informatics database queries, revising the manuscript 

HB: revising the manuscript

TH: revising the manuscript

MR: revising the manuscript

AKN: project idea, supervision, study design, revising the manuscript.

### Conflict of interest

All authors declare that they have no conflict of interest.

### Acknowledgment

We would like to thank the Federal Ministry of Economics and Climate Protection for partial financing (01MK2000G) of this project in the AIQNET consortium. Furthermore, we extend our heartfelt gratitude for the invaluable support provided by the clinical research section of the Department of Traumatology and Reconstructive Surgery at the University of Tübingen, situated at the BG Klinik Tübingen, for their diligent coordination of sample collection. Part of the data was processed within Lisa Kloppenburg's medical dissertation. 

## Figures and Tables

**Table 1:**
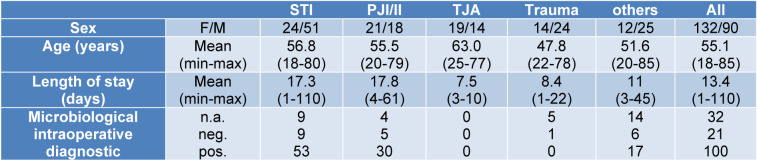
Characteristics of each group: STI=soft tissue infection, PJI/II= periprosthetic joint infection, II= implant infection, n.a.= not available

**Table 2:**
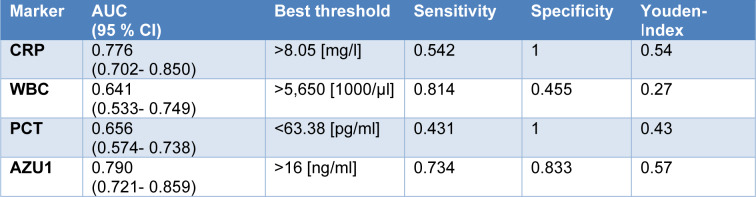
Diagnostic accuracy for CRP, WBC, PCT and AZU1 for the diagnosis of infection. CI= confidence interval

**Table 3:**
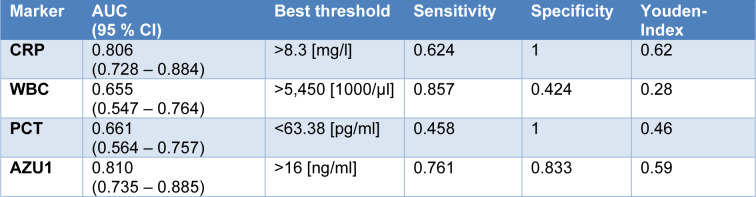
Diagnostic accuracy for CRP, WBC, PCT and AZU1 with positive microbiological samples. CI= confidence interval

## References

[1] Downes KJ, Fitzgerald JC, Weiss SL (2020). Utility of procalcitonin as a biomarker for sepsis in children. J Clin Microbiol.

[2] Fisher J, Linder A (2017). Heparin-binding protein: a key player in the pathophysiology of organ dysfunction in sepsis. J Intern Med.

[3] Funke A, Berner R, Traichel B, Schmeisser D, Leititis JU, Niemeyer CM (2000). Frequency, natural course, and outcome of neonatal neutropenia. Pediatrics.

[4] Giannoudis PV, Hildebrand F, Pape HC (2004). Inflammatory serum markers in patients with multiple trauma. Can they predict outcome?. J Bone Joint Surg Br.

[5] Halldorsdottir HD, Eriksson J, Persson BP, Herwald H, Lindbom L, Weitzberg E (2018). et al. Heparin-binding protein as a biomarker of post-injury sepsis in trauma patients. Acta Anaesthesiol Scand.

[6] Hamade B, Huang DT (2020). Procalcitonin: where are we now?. Crit Care Clin.

[7] McNally M, Sousa R, Wouthuyzen-Bakker M, Chen AF, Soriano A, Vogely HC (2021). The EBJIS definition of periprosthetic joint infection. Bone Joint J.

[8] Naito T, Jingushi K, Ueda K, Tsujikawa K (2021). Azurocidin is loaded into small extracellular vesicles via its N-linked glycosylation and promotes intravasation of renal cell carcinoma cells. FEBS Lett.

[9] Omar J, Ahmad NS, Che-Soh N, Wan-Azman WN, Yaacob NM, Abdul-Ghani NS (2023). Serum procalcitonin (PCT) - is there a role as an early biomarker in Infected Diabetic Foot Ulcer (IDFU) patients?. Malays Orthop J.

[10] Ozbay S, Ayan M, Ozsoy O, Akman C, Karcioglu O (2023). Diagnostic and prognostic roles of procalcitonin and other tools in community-acquired pneumonia: a narrative review. Diagnostics (Basel).

[11] Parvizi J, Tan TL, Goswami K, Higuera C, Della Valle C, Chen AF (2018). The 2018 definition of periprosthetic hip and knee infection: an evidence-based and validated criteria. J Arthroplasty.

[12] Pierrakos C, Vincent JL (2010). Sepsis biomarkers: a review. Crit Care.

[13] Plebani M (2023). Why C-reactive protein is one of the most requested tests in clinical laboratories?. Clin Chem Lab Med.

[14] Shohat N, Bauer T, Buttaro M, Budhiparama N, Cashman J, Della Valle CJ (2019). Hip and knee section, what is the definition of a Periprosthetic Joint Infection (PJI) of the knee and the hip? Van the same criteria be used for both joints? Proceedings of International Consensus on Orthopedic Infections. J Arthroplasty.

[15] Sigmund IK, Puchner SE, Windhager R (2021). Serum inflammatory biomarkers in the diagnosis of periprosthetic joint infections. Biomedicines.

[16] Snall J, Linner A, Uhlmann J, Siemens N, Ibold H, Janos M (2016). Differential neutrophil responses to bacterial stimuli: Streptococcal strains are potent inducers of heparin-binding protein and resistin-release. Sci Rep.

[17] Sun JK, Shen X, Sun XP, Wang X, Zhang WH, Shi QK (2020). Heparin-binding protein as a biomarker of gastrointestinal dysfunction in critically ill patients: a retrospective cross-sectional study in China. BMJ Open.

[18] Tapper H, Karlsson A, Morgelin M, Flodgaard H, Herwald H (2002). Secretion of heparin-binding protein from human neutrophils is determined by its localization in azurophilic granules and secretory vesicles. Blood.

[19] Youden WJ (1950). Index for rating diagnostic tests. Cancer.

[20] Zhou Y, Liu Z, Huang J, Li G, Li F, Cheng Y (2019). Usefulness of the heparin-binding protein level to diagnose sepsis and septic shock according to sepsis-3 compared with procalcitonin and C reactive protein: a prospective cohort study in China. BMJ Open.

